# The Role of the Ω-Loop in Regulation of the Catalytic Activity of TEM-Type β-Lactamases

**DOI:** 10.3390/biom9120854

**Published:** 2019-12-11

**Authors:** Alexey Egorov, Maya Rubtsova, Vitaly Grigorenko, Igor Uporov, Alexander Veselovsky

**Affiliations:** 1 Department Chemistry, M.V. Lomonosov Moscow State University, 3, 1, Leninskie gori, Moscow 119991, Russia; mrubtsova@gmail.com (M.R.); vitaly.grigorenko@gmail.com (V.G.); iuporov@gmail.com (I.U.); 2 Institute of Biomedical Chemistry, ul. Pogodinskaya 10, Moscow 119121, Russia; veselov@ibmh.msk.su

**Keywords:** antibiotic resistance, TEM-type β-lactamases, β-lactam antibiotics, Ω-loop, inhibitor

## Abstract

Bacterial resistance to β-lactams, the most commonly used class of antibiotics, poses a global challenge. This resistance is caused by the production of bacterial enzymes that are termed β-lactamases (βLs). The evolution of serine-class A β-lactamases from penicillin-binding proteins (PBPs) is related to the formation of the Ω-loop at the entrance to the enzyme’s active site. In this loop, the Glu166 residue plays a key role in the two-step catalytic cycle of hydrolysis. This residue in TEM–type β-lactamases, together with Asn170, is involved in the formation of a hydrogen bonding network with a water molecule, leading to the deacylation of the acyl–enzyme complex and the hydrolysis of the β-lactam ring of the antibiotic. The activity exhibited by the Ω-loop is attributed to the positioning of its N-terminal residues near the catalytically important residues of the active site. The structure of the Ω-loop of TEM-type β-lactamases is characterized by low mutability, a stable topology, and structural flexibility. All of the revealed features of the Ω-loop, as well as the mechanisms related to its involvement in catalysis, make it a potential target for novel allosteric inhibitors of β-lactamases.

## 1. Introduction

The global rise in antibiotic consumption is simultaneously increasing the number of microorganisms that have antimicrobial resistance [1]. The emergence of resistant bacteria shortens the life span of antibiotics and represents a serious challenge for modern medicine. Cephalosporins and penicillins are the most commonly used β-lactam antibiotics, and resistance toward them is also the most commonly observed [2,3]. The key mechanism of this bacterial resistance type is the hydrolysis of antibiotics by β-lactamases (βLs). Their widespread prevalence is due to the localization of the genes that encode βLs on mobile genetic elements, and, for this reason, they may be quickly transferred between bacteria [4]. βLs belong to the superfamily of enzymes that hydrolyze the β-lactam ring, and about 2800 βLs have been isolated and described from clinical bacterial strains [5]. These enzymes differ in their structure, catalytical activity, specificity, and resistance to inhibitors. They are divided into the four molecular classes of A, B, C, and D according to their primary sequence homology [6]. Class A, C, and D enzymes carry a serine residue in their active site, while class B βLs are metalloenzymes and contain one or two zinc ions. Class A βLs belong to the largest and most common group in this superfamily, which can be subdivided into enzymes of different types (including TEM-, SHV-, and CTX-M-types).

The prevalence of resistant bacteria has significantly decreased the available choices for treatment, and it has also increased the need for the development of novel antibiotics and inhibitors of βLs. The use of inhibitors, whose structures are based on the β-lactam ring, is also limited because resistance to them has also developed. Today, a promising trend is to design novel βL inhibitors and simultaneously use them with antibiotics [7,8]. Computer methods involving the in silico search of novel inhibitors has significantly broadened the range of potential inhibitors. However, only a limited number of novel βL inhibitors have been found that are of non-β-lactam nature and are capable of binding close to the enzyme’s active site [8,9,10,11]. Because of the relatively low inhibition constants of such inhibitors, this area of research needs to be further developed.

Recently, special attention has been paid to studying the role of loops and peptide linkers as flexible elements in the functioning of proteins and enzymes [12,13]. The loops, as secondary structural elements of proteins, are characterized by an enhanced mobility; their role is not solely confined to being connecting units [12]. Furthermore, changes in the amino acid composition of the loops may impart new functions to protein superfamilies. The Ω-loops, a special class of loops with a conformation resembling the Greek letter “omega,” are currently attracting specific attention. The loop conformation is ensured by the short-distance fixation of terminal amino acids. Ω-Loops have been observed in 60 proteins [13], some of which have been found to be involved in allosteric regulation during biospecific ligand recognition [14,15]. The structure of serine class A βLs represents a compact, conserved scaffold that consists of secondary structural elements linked by flexible loops. The Ω-loop is located at the bottom of the entrance to the enzyme active site and includes the catalytically important and highly conserved residue Glu166, the mutation of which leads to an almost complete loss of enzyme activity. This review focuses on the structural peculiarities of the Ω-loop of TEM-type βLs—the most versatile group of serine class A enzymes that still remain one of the most common βLs among bacterial clinical pathogens and soil bacteria [16,17]. A detailed analysis of the loop is carried out in order to establish its role as a site of the allosteric regulation of activity and the specificity of βLs, as well as a potential target for novel inhibitors.

## 2. Structural Relationship between Penicillin-Binding Proteins and β-Lactamases

**Figure 1:**
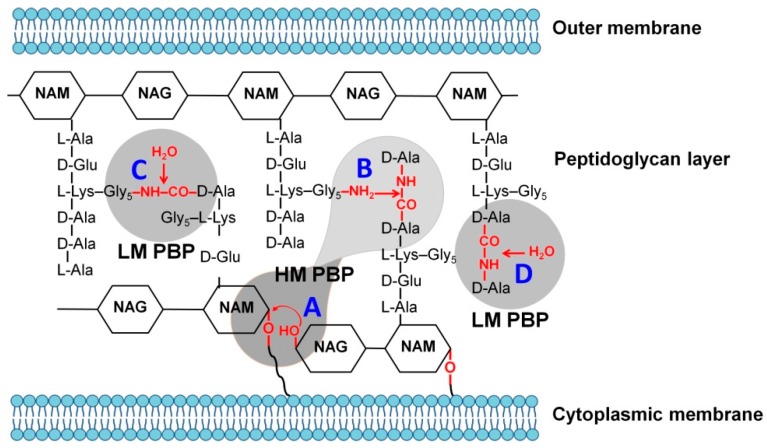
The participation of low molecular weight (LM) and high molecular weight (HM) penicillin-binding proteins (PBPs) in the synthesis of peptidoglycan. A—transglycosylation; B—transpeptidation; C—endopeptidation; and D—carboxypeptidation.

The synthesis of antibiotics by microorganisms has existed in nature for more than two billion years and is representative the struggles of species for energy sources and habitats. β-Lactams are the antibiotics that are most capable of suppressing microorganism growth by inhibiting penicillin-binding proteins (PBPs), the superfamily of enzymes involved in the diverse processes of the biosynthesis of peptidoglycan, the key structural element of bacterial cell walls [18,19]. PBPs consist of two groups of high- and low-molecular-weight enzymes that are further subdivided into classes according to the homology of their primary structure [20]. PBPs catalyze different processes that are involved in peptidoglycan synthesis, e.g., the lengthening of glycan chains (transglycosylation), cross-linking between the two glycan chains (transpeptidation), the hydrolysis of the bond between two glycan chains (endopeptidation), and the hydrolysis of the bond in D–Ala–D–Ala dipeptide (DD–carboxypeptidation) [21,22] (Figure 1). The mechanism of these reactions includes the formation of the stable acyl–enzyme complex that involves active-site serine residue of PBPs.

Due to their basic element, the β-lactam ring, β-lactam antibiotics represent a structural analogue of the D–Ala–D–Ala dipeptide and can compete with it, forming a covalent acyl–enzyme complex with the serine in the PBP active site. As a result, cell wall synthesis is inhibited, and the bacterial cell dies [23]. The expression of mutant forms of PBP (in particular PBP2a) that are characterized by a reduced affinity for β-lactams, is the reason behind the developing resistance in gram-positive bacteria [24,25].

**Figure 2:**
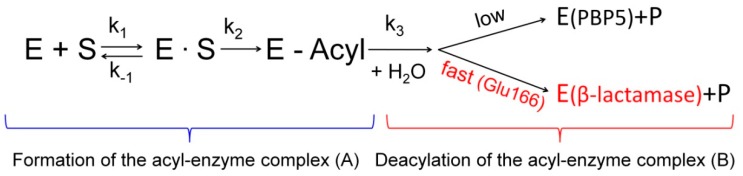
Schematic presentation of the interaction of penicillin-binding proteins and β-lactamases with β-lactam antibiotics. Both β-lactamases and PBPs form an acyl–enzyme complex. Only β-lactamases and some PBPs (for example, PBP5) can catalyze the deacylation of this complex. (**A**) Formation of the acyl-enzyme complex; (**B**) Deacylation of the acyl-enzyme complex.

The resistance of gram-negative bacteria to β-lactams is primarily caused by the synthesis of βL enzymes belonging to the EC 3.5.2.6 class of hydrolases [5,26]. A structural analysis of PBP and βL superfamilies demonstrated that they have a common ancestor that has given rise to several types of PBPs and βLs of different molecular classes in independent (and probably parallel) processes [27]. The evolutionary relationship between PBPs and βLs is that they both have a common scaffold that carries catalytically important structural motifs: Ser–X–X–Lys, Ser–X–Asn and Lys–Thr–Gly [28] and form a covalent complex between active-site serine and an antibiotic molecule (acyl–enzyme) (Figure 2A). However, unlike PBPs, class A βLs carry the Glu166 residue in the Ω-loop, so they can catalyze subsequent deacylation of this complex while recovering the active enzyme (Figure 2B).

**Figure 3:**
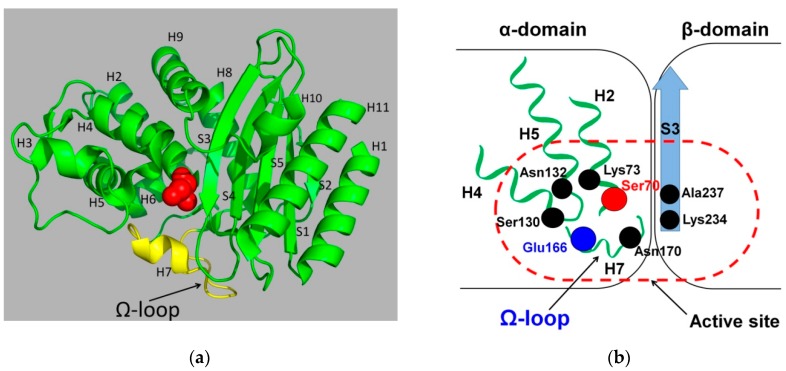
(**a**) The spatial arrangement of structural elements in the tertiary structure of β-lactamase TEM-1 (isolated from *Escherichia coli*, PDB ID 1ERO [32]). The catalytic Ser70 is shown in spherical representation, and the Ω loop is highlighted in yellow. (**b**) Scheme of the β-lactamase active site indicating the catalytically important residues and structural elements on which they are located.

In order to analyze the structural differences between βLs and PBPs, allowing the first to carry out effective deacylation, we analyzed a family of TEM-type βLs, which is the most diversified (involved in over 200 mutant forms of βL TEM-1) and actively studied the family among all class A enzymes [16,29]. The primary amino acid sequence of TEM-type enzymes consists of 286 amino acid residues, including a 23-mer signal peptide. The secondary structure forms a three-domain α/β/α sandwich complex consisting of eleven α-helices and five β-strands that are linked by loops with an irregular structure. The first and the third α-domains contain three (H1, H10, and H11) and eight (H2–H9) α-helices, respectively; the second β-domain consists of five β-strands (S1–S5) (Figure 3a) [30].

The catalytic site of TEM βLs is located between the domain with the H2 α-helix, carrying the catalytic Ser70 and β-sheet domain (Figure 3b). Residues Lys73 and Lys234, two residues (Ser130 and Asn132) of the SDN-loop residues, and two residues (Glu166 and Asn170) of the Ω-loop are involved in the catalytic cycle of hydrolysis [28,31].

## 3. Origin and Structure of the Ω-Loop in Class A β-Lactamases

Some PBPs have evolutionarily acquired an ability to slowly hydrolyze the peptide bond in D–Ala–D–Ala, which is required for regulating the length of peptidoglycan molecules. In particular, PBP5 from certain bacteria has these properties [33,34]. The hydrolytic activity of these PBPs is attributed to the emergence of two loops in the protein structure: The first loop (residues 77–87 in PBP5 from *Escherichia coli*) is involved in dipeptide orientation during acylation; the second loop (residues 147–158 in PBP5 from *E. coli*) is formed at the active site entrance and is involved in the peptide bond hydrolysis in D–Ala–D–Ala. In the course of evolution, these PBPs became the ancestors of certain βLs due to the loops’ transformations. The first loop (residues 77–87 in PBP5 from *E. coli*) was shortened to five residues (residues 101–105 in TEM-type βLs), while the second loop (residues 147–158 in PBP5 from *E. coli*) was extended to 16 residues (164–179 in TEM-type βLs). These substitutions reduced the affinity of βLs for peptidoglycan and significantly enhanced their ability to deacylate the acyl–enzyme complex (Figure 2B). Hence, these structural alterations led to the emergence of a fundamentally new function: PBPs have evolved into βL enzymes that are capable of efficiently catalyzing the hydrolysis of β-lactams.

**Figure 4:**
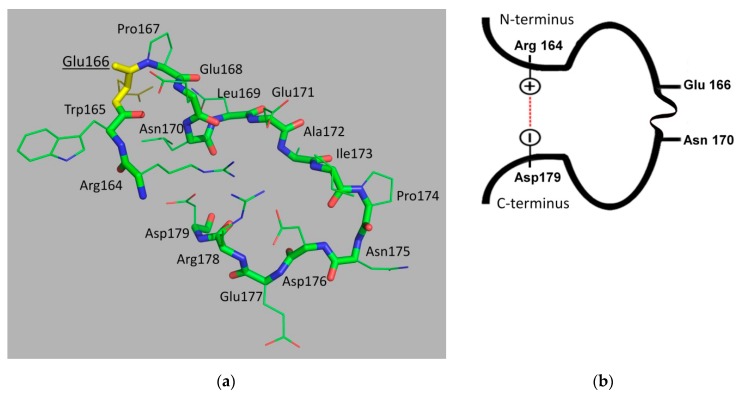
(**a**) The Ω-loop structure of β-lactamase TEM-1 (isolated from *Escherichia coli*, PDB ID 1ERO [32]). Residue Glu166 is highlighted in yellow. (**b**) Model of the Ω-loop showing the residues Glu166 and Asn170 involved in the catalytic cycle and the ionic bond between the terminal residues Arg164 and Asp179.

The extended second loop, the so-called Ω-loop (Figure 4), is present in all serine βLs of classes A, C and D. The Ω-loop of TEM-type βLs contains 16 residues (from Arg164 to Asp179) and is located at the bottom of the enzyme active site. Glu166, highly conserved in class A βLs, plays a crucial role in the hydrolysis of β-lactams [35]. The overall configuration of the loop is caused by the presence of charged and hydrophilic residues in its structure that interact both with each other and with other protein residues. Its N- and C-termini are brought closer and form a “bottleneck” as a result of multiple connections: two ionic bonds between the side-chain group of Arg164 with Asp179 and Glu171 residues, as well as the hydrogen bond between the amino group of the Arg164 main chain and side-chain group of Asp179 (Figure 4b).

All known spatial structures of TEM-type βLs have the same conformation of the N-terminal part of the Ω-loop (residues Arg164–Asn170). The conformation of this part of the loop is relatively rigid because of several points of contacts between the loop residues. Arg164 is involved in the maintenance of the loop structure with a “bottleneck,” as described above. Trp165 interacts with Leu169. Glu166 is a catalytic residue involved in the two-step hydrolysis of antibiotics. The cis-configuration of the peptidyl-proline bond (Glu166–Pro167) is a prerequisite for the catalytically active orientation and formation of a hydrogen bonding network involving Glu166, Asn170, and a water molecule [36,37]. These residues are located close to each other due to the short α-helix formed by Pro167–Asn170 residues.

The C-terminal part of the Ω-loop (from Glu171 to Asp179) is a relatively flexible element of the protein globule. The side-chain of Glu171 is turned inward to the loop and forms ionic bonds with the residues Arg164 and Arg178 at its N- and C-termini. The residues Ile173 and Asp176 form a hydrogen bond and give rise to a β-rotation in this part of the loop; the side-chain group of Asp176 is inward-oriented, as it forms an ionic bond with Arg178 and a hydrogen bond with the backbone of the Asp163 and Arg164 residues. The side-chain groups of the Ala172, Pro174, and Asn175 residues do not interact with other amino acid residues.

In addition to being involved in inter-residue interactions inside the Ω-loop, its amino acid residues interact with other structural elements of the protein globule. Two amino acid regions which have an elongated conformation, the Val159–Asp163 region and the short β-strand Thr180–Pro183, are located immediately before and after the Ω-loop, respectively. The N-terminus of the loop is fixed by a peptide consisting of nine residues (155–163) and is a part of the H6 α-helix. They form several hydrogen bonds with the loop residues (Arg161–Asp179, Arg161–Thr180, and Asp163–Asp179). The Thr180–Pro183 region continues the amino acid sequence of the C-terminal part of the loop and is a short β-strand lying parallel to another short β-strand (Arg65–Met68) whose resides are located near the N-terminus of the Ω-loop. These secondary structural elements are interconnected by hydrogen bonds between the Asp179–Met68, Thr181–Phe66 and Thr180–Arg65 residues and an Arg65–Glu177 ionic bond, all of which stabilize the conformation of the loop. The interaction between the N- and C-terminal parts of the Ω-loop and the adjacent protein regions results in the formation of a structure that resembles a β-strand consisting of three oppositely oriented strands stabilized by a hydrogen bonding network. The interaction between the Ω-loop residues and other amino acid residues of the protein is caused by hydrophobic contacts between Trp165 and the Leu139 and Pro145 residues. The loop residue Glu166 forms an ionic bond with the catalytically important Lys73 residue.

Studies focused on the behavior of water molecules located inside the cavity under the Ω-loop have demonstrated that they form three dynamically stable water bridges with the residues of the loop and the protein globule [38]. Their arrangement is consistent with the crystallization water molecules in the known spatial structures of βL TEM-1. This is probably why the N-terminal part of the loop is rigid but its C-terminus is mobile.

**Figure 5:**
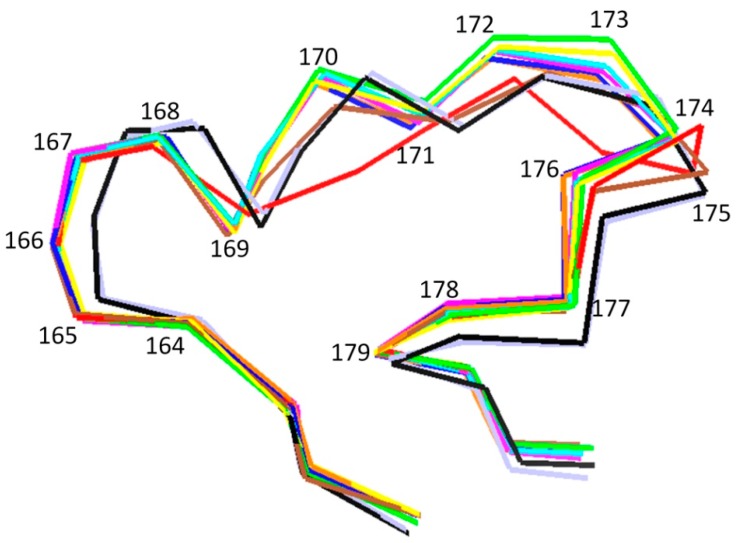
The superposition of the spatial structures of Ω-loops of class A β-lactamases in trace presentation, TEM-1 (green), TEM-64 (red), SHV-1 (yellow), CTX-M-96/12 (violet), CTX-M-44 (blue), TOHO-1 (black), PSE-4 (orange), KPC-2 (cyan), L2 (brown), SFC-1 (light-blue).

In order to compare the Ω-loop conformation of enzymes belonging to class A βLs, the corresponding spatial structures of ten enzymes (including TEM-, SHV-, CTX-M-, KPC-types, and some others) were analyzed (Table 1). Though the amino acid sequences have been found to partially differ in all the studied enzymes, the conformational topology of the Ω-loop has been found to be similar (Figure 5). This phenomenon can be explained by the spatial arrangement of the terminal loop residues Arg164 and Asp179: The distance between them is virtually unchanged between the different enzymes, since the loop starts and ends in the stable region of the protein, which is characterized by low flexibility caused by the interplay between the secondary structural elements.

## 4. The Role of the Ω-Loop in the Catalytic Cycle of TEM Type β-Lactamases

**Figure 6:**
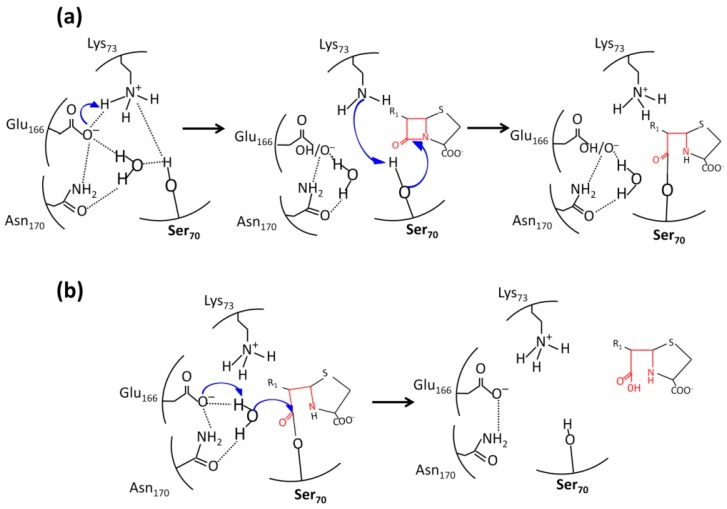
The participation of Glu166 and Asn170 of the Ω-loop of in the catalytic cycle of TEM-type β-lactamases. (**a**) Acylation of the catalytic Ser70; (**b**) deacylation of the acyl–enzyme complex.

The mechanism of catalytic action of βLs belonging to classes A, C, and D is similar to that of serine proteases (two-step acylation/deacylation). The detailed mechanism of enzymatic hydrolysis was elucidated using the data on the crystal structures of class A βLs with a resolution <1 Å and quantum molecular dynamics simulations [25,48]. This mechanism involves the consecutive stages: antibiotic binding, giving rise to the preacylation complex, then a covalent acyl–enzyme complex with catalytic serine 70 is formed, followed by the deacetylation stage (Figure 6).

In the apo form of TEM type βL, the Ω-loop residue Glu166 is deprotonated and forms an ionic bond with Lys73 that is protonated and positively charged (Figure 6a) [35]. A hydrogen bonding network is formed that involves an active site water molecule and the Glu166 and Asn170 residues of the Ω-loop [49]. When the enzyme binds to an antibiotic molecule, the distribution of charged residues in the active site varies: The Lys73 residue becomes deprotonated and performs a nucleophilic attack on Ser70, causing its deprotonation and interaction with the carbonyl carbon atom of the β-lactam ring of the antibiotic in order to give rise to the covalent acyl–enzyme complex. Studying the water bridges between the Ω-loop residues Glu166 and Asn170 and the catalytic residue Ser70 revealed that the retention time of a water molecule is approximately 500 ps [50]. This water molecule is considered to be a proton bypass between Glu166 and Ser70.

In the second stage (Figure 6b), Glu166 deprotonates the water molecule, which attacks the carbonyl atom of the antibiotic. The bond of this atom with oxygen atom of the Ser70 in the acyl–enzyme complex is destroyed, leading to the release of the enzyme and hydrolyzed antibiotic molecules.

The role of Glu166 as a common base in proton transfer during the formation and, especially, the degradation of the acyl–enzyme complex can be explained by a change in its location relative to other catalytic residues and the antibiotic molecule due to the mobility and flexibility of the Ω-loop. This was experimentally confirmed when this residue was substituted for either cysteine or aspartic acid, causing the deacylation rate in mutant enzymes to decrease by several orders of magnitude [51]. Hence, the presence of the Glu166 residue in the Ω-loop fundamentally distinguishes class A βLs from PBPs, and this residue plays a crucial role in the two-step hydrolysis of β-lactam antibiotics.

## 5. The Effect of Mutations in Ω-Loop Residues on Properties of TEM Type β-Lactamases

**Figure 7:**
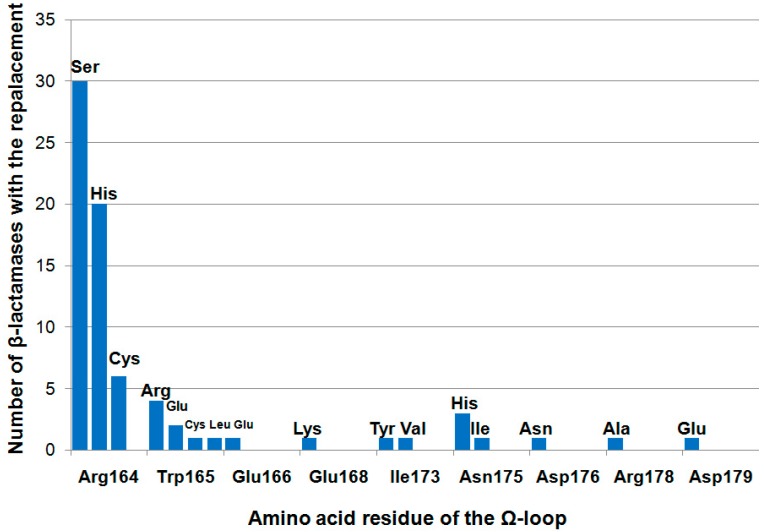
The frequency of mutations of Ω-loop residues in TEM-type β-lactamases that are isolated from clinical bacterial strains.

The TEM-type βL family is characterized by a high degree of mutability (~30% of amino acid residues were found to mutate). Some of the mutations affect the substrate specificity, enzyme activity, and thermal stability [16,17]. At the same time, the Ω-loop is a highly conserved region (Figure 7). The most frequently mutated residue is Arg164; its substitutions occur in 56 enzymes that are isolated from clinical bacterial strains. The Trp165 residue mutates significantly less frequently: Its mutations were revealed in nine enzymes. Mutations in other residues are found extremely rarely in βLs that are clinically relevant.

The Arg164 residue is most often replaced by serine (30 enzymes), histidine (20 enzymes), and, less frequently, by cysteine (six enzymes). All the Arg164Ser/His/Cys substitutions are functionally important (the “key” ones) because they give rise to extended spectrum βLs (ESBLs) that can hydrolyze not only penicillins and first-generation cephalosporins but also the second-to-fourth generation oxyimino-cephalosporins. The Arg164His and Arg164Cys substitutions disrupt the ionic bonding between the two terminal Ω-loop residues (His/Cys164 and Asp179), thus increasing the volume of the active-site cavity to bind the cephalosporins with bulky side-chain substituents [39,52].

The Arg164Ser substitution retains the hydrogen bond between Ser164 and Asp179 [53], which additionally stabilize the Ω-loop conformation. This explains why this replacement is the most common among clinical strains as the producers of TEM-type βLs. Regarding mutant enzymes with the Arg164Ser substitution, the catalytic efficiency (k_cat_/K_M_) of cefotaxime and ceftazidime hydrolysis increases 5- and 200-fold, respectively, compared to that for the wild-type TEM-1 enzyme; the efficiency of ampicillin hydrolysis is simultaneously significantly reduced [52,54]. It is expected that Arg164Ser and Arg164His mutations can lead to a more rapid water molecule exchange near the Ω-loop, contributing to the destabilization of the loop [38].

The hypothesis that enzymes carrying a mutation of the Arg164 residue have a limited number of active site conformation states, thus providing an advantage for efficient antibiotic binding and hydrolysis, is an alternative explanation as to why the activity of mutants toward bulky oxyimino-cephalosporin molecules increases [55]. Contrariwise, the wild-type βL TEM-1 is characterized by a large set of nonproductive active-site conformations.

The Trp165 residue is substituted for one of the four amino acids (Arg, Cys, Glu, or Leu) and is the only mutable tryptophan residue among the four tryptophan residues in TEM-type βLs [56]. The Trp→Arg substitution leads to a redistribution of the contacts between the Ω-loop residues, and a new ionic bond is formed between Arg165 and Glu168; this bond alters the enzyme’s ability to bind to β-lactams. In particular, TEM βLs become unable to interact with clavulanic acid and eventually develop a resistance to this inhibitor [57].

Studies focused on βLs with substitutions of Glu166Ala, Glu166Cys, and Glu166Asp, which are artificially synthesized, have demonstrated that the substrates exhibit a good binding to the active site of the enzymes, but their hydrolysis rate is significantly reduced [51]. As a result, the catalytic efficiency of hydrolysis decreases by six, five, and three orders of magnitude, respectively. A similar effect of catalytic activity reduction by six orders of magnitude has been demonstrated for the Glu166Tyr substitution in combination with the Trp165Tyr and Pro167Gly substitutions [58]. All mutants that carry a single Glu166 substitution or its combination with other substitutions in the Ω-loop have been found to be characterized by a lower K_M_ of oxyimino-cephalosporins compared to those of the wild-type enzyme and the enzyme carrying single mutations at positions 165 and 167. The fact that the binding of antibiotics with bulky substituents is the most efficient example has been attributed to conformational changes in the Ω-loop and other regions of the protein globule, which increased the volume of the active-site cavity [29,58].

Randomized mutant libraries of TEM-type βLs have been produced to study the individual effect of artificial mutations in the residues of the Ω-loop on the substrate specificity of the enzymes [59,60]. The substrate specificity was determined according to the minimum inhibitory concentrations (MICs) of ampicillin and ceftazidime. The activity of βLs against ampicillin has been found to be extremely sensitive to mutations in the Ω-loop. The enzyme stays highly active if the Arg164, Glu166, Pro167, Asn170, Asp176, and Asp179 residues are conserved and mutual contacts (Arg164–Asp179 and Asp176–Arg178) are retained; the Arg178 residue can be substituted only for amino acids whose side chains can donate protons. It has been revealed that individual Ω-loop residues do not need to remain conserved in ceftazidime-resistant mutants. It seems that Ω-loop destabilization and the disruption of contacts between its residues are required to ensure the efficient hydrolysis of cephalosporins. In this case, loop mobility is improved, so bulky cephalosporin molecules have a better orientation in the active site. The ceftazidime-hydrolyzing mutants have, in most cases, been characterized by a lower expression level and a reduced stability compared to the wild-type enzyme.

## 6. The Molecular Dynamics of the Ω-Loop of Class A β-Lactamases and Its Mobility

The molecular dynamics simulations of TEM type βLs have demonstrated that the Ω-loop mobility varies, depending on conditions and enzyme type [44,47,61,62]. An investigation of βL TEM-1 and its mutants that carry two key mutations (Gly238Ser and Glu240Lys) and two secondary mutations (Met182Thr and Gln39Lys) showed that each mutation has its own effect on the mobility of the Ω-loop [62]. The key mutations have been found to increase the amplitude of loop movements; the additional secondary mutation Met182Thr has been found to stop the movements of the C-terminal part of the loop and to cause its fixation near the protein globule, while the secondary mutation Gln39Lys makes the loop mobile again. An investigation of alterations in amino acid contacts of the enzyme with a combination of the key mutations and the secondary mutation Met182Thr demonstrated that the Ω-loop position changes depending on the conformation of the Arg65 residue [63]. The C-terminal part of the Ω-loop of this mutant is fixed near the protein globule because of the weakened interactions in the hydrophobic patch that carries the mutable residues; as a result, Arg65 changes its position and interacts with the Asn175 and Asp176 residues [61,63]. A similar effect was observed earlier [38] by using long molecular dynamics simulation times (up to 20 ns). A hypothesis was put forward that this fixation of the Ω-loop may promote the proper positioning of the Glu166 residue for catalytic activity [64].

NMR studies have revealed the increased mobility of two residues (Arg161 and Arg178) at the N- and C-termini of the Ω-loop in βL PSE-4 [64]. It has been assumed that this may lead to the contact between Gly175 (an equivalent of Asn175 in βL TEM-1) and Arg65. A principal component analysis of the cooperative motion of the Ω-loop revealed an additional two orientations of synchronized motion; however, the functional significance of these motions still needs to be elucidated [61].

Substrate binding in the active site of the βLs TEM-1 and PSE-4 increases the mobility of their Ω-loops, which may be attributed to the fact that the loop is involved in substrate delivery to the active site [61].

Short molecular dynamics trajectories for the enzyme that carries the Met69Leu mutation responsible for resistance to βL inhibitors have shown changes in Ω-loop mobility [65]. The binding of the protein inhibitor to βL SHV-1 and its Trp229Ala mutant has been shown to enhance the fluctuations of the Ω-loop [66].

Hence, an analysis of the mobility of the Ω-loop demonstrated that it is rather flexible. In the conformational states of the loop under analysis, the enhanced rigidity of the N-terminal part of the loop is combined with the high mobility of its other fragments. The structural rigidity of this part of the loop, which carries the Glu166 residue, is needed for the accurate positioning of the substrate and catalytic residues, whereas the mobility of the other part of the loop is needed to ensure substrate delivery to the active site and the fine-tuning of the catalytic cycle of hydrolysis.

## 7. Current Approaches for Overcoming the Resistance Conferred by β-Lactamases

The conventional approach to resistance suppression is based on inhibitors that carry the β-lactam ring; these inhibitors form stable acyl–enzyme complexes with catalytic Ser 70 [67,68]. Such inhibitors are clavulanic acid, sulbactam, and tazobactam, all of which are used in clinical practice in combination with antibiotics. The hydrolysis rate of βL–inhibitor complexes is several orders of magnitude lower than that of the βL–antibiotic complexes. However, there are only few efficient inhibitors based on the β-lactam structure, and they all have a narrow specificity [69]. Furthermore, the wide use of inhibitors has led to the emergence of mutant forms of βLs that can deacylate enzyme–inhibitor complexes and are therefore inhibitor-resistant. A new strategy is currently under development for discovering potential inhibitors that target the active site but do not carry the β-lactam ring [8]. Of these, compounds belonging to the diazabicyclooctane (DBO) class have been proven to be most efficient. This inhibitors include avibactam and relebactam, the agents forming carbamyl–enzyme complexes with the catalytic serine residue, which further undergo slow, reversible recyclization with an inhibitor molecule being released [70,71]. These inhibitors exhibit activity against βLs of classes A, C, and, partially, against class D [72,73]. However, microorganisms also develop resistance to these inhibitors [74,75]. Boronic acid derivatives are also being actively studied as novel inhibitors of βLs [76]. In terms of their mechanism of action, these compounds are transition-state inhibitors, since their complex with the enzyme has a tetrahedral conformation that mimics the transition state of the tetrahedral oxyanion in acylation and deacylation reactions [77]. A fixed-dose combination of meropenem and vaborbactam (a monocyclic boric acid derivative), an inhibitor of class A and C βLs, is used in clinical practice [78,79].

A new direction involves searching for structural analogues of known βL inhibitors, including allosteric ones. The computational ligand-based in silico screening technique, in combination with molecular docking and experimental studies of βL inhibition, has been applied for the identification of acylated phenoxyaniline and thiourea compounds as novel non-β-lactam inhibitors of TEM-type βLs [11,80]. An analysis of the residues involved in the binding of the inhibitor at the active site of the enzyme showed that some of them are conservative for class A βLs, which can be prospective for inhibitors of these enzymes.

The Ω-loop of class A βLs may also be considered as a target for inhibition. Blocking the key Ω-loop residues included in the catalytic cycle prevents deacylation and the release of free enzyme molecules. Located in the external structure of the globule and accessible to a solvent, the loop enables exposure to it from the outside of the protein. This is indicated by data that have shown that the Ω-loop is highly immunogenic [81]. A typical feature of the Ω-loop of TEM-type βLs is that its N-terminal part is rigid, while its C-terminal part is relatively flexible and is responsible for the required orientation of Glu166 with respect to the active site during the hydrolysis. Inhibition can be expected to occur if Glu166, the key residue of the Ω-loop, changes its orientation or position due to the interaction of the inhibitor with the loop. On the basis of the structural and functional peculiarities of the Ω-loop, it can be considered to be a “mosaic” model of targets for allosteric inhibitors.

## 8. Conclusions

As a result of the increase in the consumption of β-lactams in the 21st century, the problem of overcoming bacterial resistance has become extremely urgent. β-Lactam antibiotics represent the most widely used antimicrobials, and in accordance with current assessment, their consumption will have doubled by 2030 [1]. Resistance to them is the most widespread worldwide, and is conferred by the production of βLs, representing a superfamily of bacterial enzymes. This makes the development of βL inhibitors based on novel principles and chemical structures especially relevant.

A novel approach to the development of βL inhibitors is based on the search for “hot spots,” which are the centers of allosteric regulation, in the protein globule. Some of the “key” drug resistance mutations that influence enzyme structure and activity are found in the loops. Therefore, their localization sites can be regarded as potential targets for inhibitors. Special attention should certainly be paid to the loops that surround the active site and that affect the activity and specificity of βLs.

The analysis of the Ω-loop performed in this review demonstrates that it can become a novel target for designing allosteric inhibitors of class A βLs. Once evolved, the βLs acquire a new function—the ability to hydrolyze the acyl–enzyme complex due to the highly conserved Glu166 residue. This residue is involved in all stages of the catalytic cycle of hydrolysis, playing an especially important role at the stages of deacylation and the release of the hydrolyzed antibiotic molecule. All serine βLs have this loop, which is located at the active-site entrance and is oriented towards the external environment. It has a conserved topology in different class A βLs and has been shown to be highly immunogenic. It is of importance that the Ω-loop provides fine-tuning of antibiotic binding to the active site and its subsequent hydrolysis. On the basis of these properties, the Ω-loop can be regarded as the site of activity regulation of these βLs, the most common enzymes in clinically relevant bacteria, which induce a broad antimicrobial resistance.

All the aforementioned features of the Ω-loop allow one to consider it a promising target for designing a family of different inhibitors of class A, C, and D βLs with allowance for the peculiarities of the Ω-loop’s structure, the type of β-lactam antibiotic, and the type of a microorganism.

Designing novel allosteric βL inhibitors and their uses in combination with antibiotics will lengthen the life span of β-lactams whose therapeutic action has been well studied and has advantages such as a broad specificity, a low toxicity, and a low allergenicity compared to other antibiotics.

## Figures and Tables

**Table 1:** The sequences of the Ω-loops of different serine class A β-lactamases.

β-Lactamase	PDB ID	Amino Acid Sequence of the Ω-Loop (Residues from 164 to 179, Glu166 is Highlighted in Bold)
TEM-1	1ERO [32]	RW**E**PELNEAIPNDERD
TEM-64	1JWZ [39]	SW**E**PELNEAIPNDERD
SHV-1	1VM1 [40]	RW**E**TELNEALPGDARD
CTX-M-12	3ZNY [41]	RT**E**PTLNTAIPGDPRD
CTX-M-44	4X69 [42]	RT**E**PTLNTAIPGDPRD
Toho-1	2ZQA [43]	RTAPTLNTAIPGDPRD
Pse-4	1G6A [44]	RI**E**PDLNEGKLGDLRD
Kpc-2	3DW0 [45]	RW**E**LELNSAIPGDARD
L2	5NE2 [46]	RL**E**PELNSFAKGDPRD
Sfc-1	4EQI [47]	RW**E**LELNSAIPGDDRD

## Abbreviations

βL—β-lactamase, PBP—penicillin-binding protein
